# Adjuvant Metronomic Chemotherapy After Surgery in pT1-T2 N0 M0 HER2-Positive and ER/PR-Positive Breast Cancer Plus Targeted Therapy, Anti-Hormonal Therapy, and Radiotherapy, with or Without Immunotherapy: A New Operational Proposal

**DOI:** 10.3390/cancers17081323

**Published:** 2025-04-15

**Authors:** Luca Roncati

**Affiliations:** Department of Life Sciences, Health, and Health Care Professions, Link Campus University, 00165 Rome, Italy; l.roncati@unilink.it or emailmedical@gmail.com

**Keywords:** breast cancer, sentinel lymph node biopsy, metronomic chemotherapy, human epidermal growth factor receptor 2 (HER2), estrogen receptor, progesterone receptor, targeted therapy, trastuzumab, antihormonal therapy, immunotherapy

## Abstract

Breast cancer is the most common and deadly female-specific malignancy in the world. Four immunohistochemical subtypes are distinguished: luminal A, luminal B, HER2-positive, and triple-negative. In turn, the HER2-positive subtype presents two variants depending on the status of the hormone receptors. The variant that expresses them can benefit from both anti-HER2 and anti-hormonal therapy. Today, MCTP finds application in maintenance therapy after standard of care and in advanced breast cancer when the patient’s clinical condition is already seriously compromised by metastatic disease; in this context, it is used as a first-line treatment, in pre-treated subjects, or as a rescue treatment. Here, the use of adjuvant oral MCTP after surgery at an early stage in HER-2 and hormone-positive local breast cancer is proposed, where effective treatment options are available, such as anti-HER2 therapy (e.g., trastuzumab, pertuzumab), anti-hormonal therapy (e.g., tamoxifen, letrozole), radiotherapy, and, in case of strong PD-1 positivity, immunotherapy.

## Simple Summary

Metronomic chemotherapy (MCTP) consists of frequently administering low doses of chemotherapy to reduce its side effects, without extended drug-free breaks. Here, its oral adjuvant use after surgery in combination with targeted therapy, anti-hormonal therapy, and radiotherapy is proposed in a variant of breast cancer smaller than 5 cm, not metastatic to the lymph nodes or elsewhere, and expressing both human epidermal growth factor receptor 2 (HER2) and hormone receptors. The possible improvement with immunotherapy using monoclonal antibodies against programmed death 1 (PD-1) is also considered.

## 1. Metronomic Chemotherapy

Metronomic chemotherapy (MCTP) is an innovative approach to treat cancer based on the continuous administration of known chemotherapy drugs at doses lower than the maximum tolerated ones, but for a longer period of time, in order to reduce their side effects [1,2,3,4]. It is commonly administered orally, which is more convenient and comfortable for patients than the intravenous route usually used in conventional chemotherapy, where the drug is administered at a dosage close to the maximum tolerated dose in bolus mode to obtain the cytotoxic effect on tumor cells [5]. However, by doing so, even the healthy, fast-dividing cells of the body are affected, e.g., bone marrow cells, epithelial cells in the gastrointestinal tract, and hair stem cells, causing more or less significant side effects such as cytopenia, nausea, vomiting, diarrhea, and hair loss; a break in treatment is therefore necessary to allow these normal cells to recover [1,2,3,4,5]. Because in MCTP a much lower dosage of the drug, usually one-tenth to one-third of the maximum tolerated dose, is frequently administered to maintain a low concentration in the plasma, the probability of serious side effects is significantly reduced [6].

At a metronomic concentration, the drug acts mainly on the tumor microenvironment, including immune cells and tumor endothelial cells, rather than with a cytotoxic mechanism. More specifically, MCTP has been found to selectively inhibit regulatory T cells (Tregs) and thereby activate helper and cytotoxic T cells responsible for cancer-specific immunity, as well as natural killer cells involved in the innate immune response [7]. In fact, Tregs control the activity of effector T-cells and other immune cells primarily through cell-to-cell contact, as well as by producing suppressive cytokines (e.g., interleukin-10, transforming growth factor-β) [8]. This immunomodulation of Tregs is not a prerogative of conventional chemotherapy, which instead tends to reduce the number of all lymphocyte subsets [9,10]; furthermore, some chemotherapeutic agents such as cyclophosphamide, etoposide, methotrexate, paclitaxel, and vinblastine can promote, at low concentrations, the maturation and antigen-presenting ability of dendritic cells, which in turn facilitate T-cell-mediated immunity against cancer [11]. T cell activity has also been found to be potentiated by MCTP via intracellular production of type I interferon in tumor cells due to the triggered mitochondrial dysfunction [12]. Normalization of the tumor microenvironment enhances the therapeutic effect of immunotherapy by checkpoint inhibitors, which target programmed death 1 (PD-1) [13,14]. PD-1 is a lymphocyte surface receptor that downregulates the immune system by promoting self-tolerance; however, several cancers highly express PD-1 ligand (PD-L1) to evade immune recognition and escape T cells [15]. Therefore, PD-1/PD-L1 blockade increases immune activity against those breast tumors that express them [16,17]. Furthermore, MCTP induces apoptosis and inhibits the proliferation of tumor endothelial cells without disrupting the endothelial cells of normal blood vessels [18]; this is likely due to an increase in the expression of thrombospondin-1 [19]. Another target of MCTP is the bone marrow-derived circulating endothelial progenitor cells, which are involved in cancer angiogenesis and whose levels are reduced by MCTP [20,21]. Neoangiogenesis is also counteracted by decreasing circulating levels of vascular endothelial growth factor [22,23,24].

Some authors have hypothesized that the tumor response to MCTP is not limited to antiangiogenic and immunomodulatory activities but is also expressed through a Drug-Driven Dependency Deprivation, the so-called “4D” effect [25]. During prolonged exposure to chemotherapeutic agents, some cancer cells may become drug-dependent, and their growth may be significantly inhibited until cell death once the drug is removed [25]. This effect might not be sufficient to induce tumor regression, but it could lead to the destruction of the most resistant cancer clones [25].

Last but not least, MCTP may also induce tumor cell dormancy and quiescence with mechanisms still under investigation. Dormancy is a cancerous state of variable duration in which tumor cells cease replicating but continue to survive, waiting for adequate environmental stimuli to begin proliferating again [26]. These dormant cells can remain in this state for years as minimal residual disease or isolated in the bone and may be clinically undetectable until they give rise to difficult-to-treat macrometastases [27]. Although still poorly understood, tumor collapse and hypoxia are proposed mechanisms underlying cancer dormancy [28,29,30]. The former is reached when the main tumor mass is significant and such that it has no more physical space to grow, triggering a cell contact inhibition [31], while the latter, strictly connected to the previous one, is favored by a reduced blood supply and by the loss of intratumoral vascularization inhibited precisely by MCTP [32]. A state of quiescence can also be found in micrometastases kept under control by the hosting lymph node; when this is exhausted, the tumor cells can expand and spread to distant locations via the bloodstream [33]. By inducing unresectable malignant cells into growth arrest, MCTP can allow the patient to survive longer in a chronic paucisymptomatic condition and, theoretically, make cancer a chronic disease [34,35].

## 2. Breast Cancer

**Figure 1:**
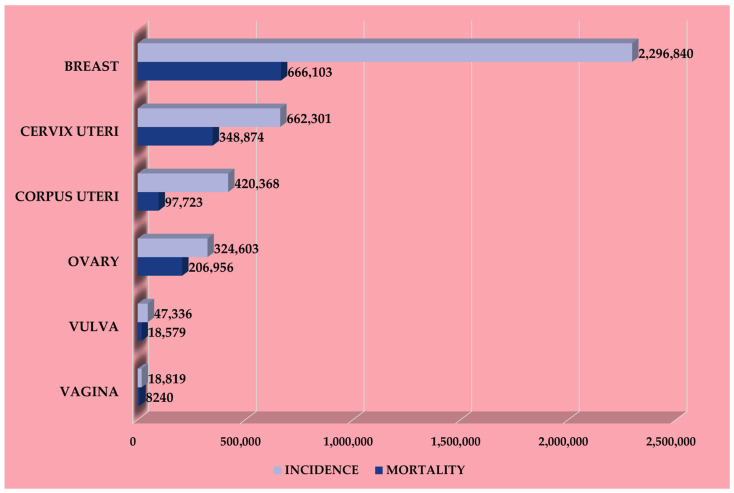
Global incidence and mortality of female tumors throughout 2022: breast cancer ranks first in both incidence and mortality. The number of deaths from breast cancer is slightly higher than new cases of cervical cancer, the second most common female tumor in the world. These data are very illustrative of the socio-health burden linked to breast cancer even today [data source: Globocan].

According to the Global Cancer Observatory powered by the International Agency for Research on Cancer of the World Health Organization, breast cancer is the most frequent worldwide among the female population and has the highest mortality rate (Figure 1).

In 2022 there were 2,296,840 new cases and 666,103 related deaths globally, with the highest mortality in Asia (47.3%), followed by Europe (21.7%), Africa (13.7%), Latin America and the Caribbean (9.0%), Northern America (7.5%), and Oceania (0.8%) [36]. Unfortunately, Asia and Europe also dominate the 2022 incidence and 5-year prevalence rankings with 985,817 and 3,197,043 cases and 557,532 and 2,296,495 cases, respectively, followed by Northern America (306,307 vs. 1,332,343), Latin America and the Caribbean (220,124 vs. 725,017), Africa (198,553 vs. 507,659), and Oceania (28,507 vs. 119,836) [36].

Over the years the surgical approach to breast cancer has been refined with skin-sparing mastectomy and quadrantectomy plus radiotherapy, and again with the introduction of sentinel lymph node biopsy (SLNB) [37]. At the beginning of the 21st century, Umberto Veronesi’s team demonstrated that axillary dissection could be safely avoided in women with invasive breast tumors 2 cm or less if SLNB was negative, thus benefiting from less pain, less swelling, and greater arm mobility [38,39]. Today, SLNB technique is standardized and routinely used in the staging of many malignancies, breast cancer included [40]. According to the pathological tumor-node-metastasis (pTNM) staging system, tumors 2 cm or less are categorized as T1, between 2 cm and 5 cm as T2, more than 5 cm in greatest dimension as T3, while tumors of any size with direct extension to the chest wall and/or to the skin in terms of ulceration or macroscopic nodules are designated T4 [41]. No metastasis in SLNB is classified as pN0, 1–3 positive axillary lymph nodes as pN1, 4–9 as pN2, ≥10 as pN3; furthermore, M0 and M1 indicate, respectively, the absence of distant metastases and clinical or radiographic evidence of distant metastases, respectively [41]. Grading (G) ranges from well-differentiated (G1) and moderately differentiated (G2) to poorly differentiated (G3) forms, almost all of which derive from the epithelium of the ducts or lobules of the breast, hence the names ductal or lobular carcinoma [41]. Rarer histotypes include mucinous, tubular, medullary, papillary, and metaplastic carcinoma [41]. In addition to morphology, immunohistochemical classification has also gained consensus for its prognostic relevance, allowing breast cancer to be divided into four molecular subtypes that share microarray signatures.

### 2.1. Luminal A Subtype

This subtype highly expresses estrogen receptors (ER) and progesterone receptors (PR) but does not express human epidermal growth factor receptor 2 (HER2). It is usually a G1 or G2 breast tumor and shows a low proliferation rate on immunohistochemistry for Kiel antigen 67 (Ki-67) [41]. Luminal A cancers generally have a poor response to conventional chemotherapy but an excellent response to anti-hormonal therapies, conferring a favorable prognosis [41]; these include selective ER modulators with antagonistic endocrine functions on the mammary gland (e.g., tamoxifen, toremifene, and raloxifene), aromatase inhibitors, which block the aromatization of androgens into estrogens by inhibiting the aromatase enzyme in a reversible (e.g., letrozole, anastrozole) or irreversible (e.g., exemestane) manner, and gonadotropin-releasing hormone analogs (e.g., triptorelin, goserelin, and leuprorelin) [42].

### 2.2. Luminal B Subtype

This subtype poorly expresses ER/PR and does not express HER2. It is usually a G3 breast tumor and shows a high proliferation rate on Ki-67 immunohistochemistry. In practice, luminal B cancers respond poorly to antihormonal drugs, and, therefore, they are burdened by an unfavorable prognosis [41]. Several classes of chemotherapeutics can be used for their treatment, such as anthracyclines (e.g., doxorubicin, epirubicin), antimetabolites (e.g., fluorouracil, capecitabine, and methotrexate), DNA alkylating agents (e.g., cyclophosphamide), platinum-based drugs (e.g., cisplatin, carboplatin), and taxanes (e.g., docetaxel, paclitaxel) [43,44]. Chemotherapy reduces the risk of cancer recurrence by about one-third over the next ten years; however, 1–2% of patients undergoing chemotherapy experience permanent or life-threatening side effects.

### 2.3. HER-2 Positive Subtype

This subtype shows HER2 amplification and may or may not express ER/PR. It is usually a G3 breast tumor and shows a high proliferation rate on Ki-67 immunohistochemistry or mitotic count [41]. Prior to the introduction of anti-HER2 therapy, this subtype was the most aggressive and characterized by the shortest survival despite chemotherapy; nowadays, thanks to HER2-targeted treatment, a significantly better prognosis has been achieved [41]. It is based on the administration of monoclonal antibodies, among which are trastuzumab and pertuzumab. Through specific binding to different epitopes, both antibodies prevent HER2 dimerization, block intracellular signaling, and cause tumor cell cycle arrest [45]. The antibody binding also induces the immune system to kill cancer cells through an antibody-dependent cell-mediated cytotoxicity [46].

### 2.4. Triple-Negative Subtype

This subtype, also called basal-like because it is thought to originate from basal cells, does not express either HER2 or ER/PR. For this reason, it is the most difficult to treat and is burdened by the highest mortality rate [41]. Typically, it is a fast-growing G3 breast cancer that occurs in the context of mutations in the breast cancer gene 1 (BRCA1) [41]. About 40% of triple-negative breast cancers express androgen receptors and may respond to antiandrogen medications such as bicalutamide; efforts are underway to use it as a prognostic marker and a treatment [47]. Great hope is also placed in immunotherapy: a breast cancer is considered PD-1/PD-L1-positive if it shows any extent of membrane immunohistochemical staining in ≥1% to <49% of tumor cells, while it can be considered strongly positive and enjoys a greater therapeutic response if such staining involves ≥50% of tumor cells [48].

## 3. State of the Art

Currently, the main fields of application of MCTP in breast cancer are advanced metastatic disease from luminal B, HER2-positive, or triple-negative subtypes [49,50,51,52,53,54,55,56,57,58,59,60], in an attempt to chronicize it and prolong patient survival; maintenance therapy after conventional chemotherapy; and salvage treatment [61,62]. In this last context, it is used both in the first line and in patients pretreated conventionally [63,64,65]. The low dosages make MCTP successfully tolerable even in the elderly and in heavily pretreated subjects [66,67,68,69]. Among the drugs tested over the years, there are fluorouracil [70], methotrexate [70,71,72], docetaxel [73,74], paclitaxel [75], doxorubicin [76], eribulin [77], etoposide [78,79], temozolomide [80], gemcitabine [14], vinorelbine [81,82,83,84], cyclophosphamide [85,86,87,88], and capecitabine [89,90,91,92]. These last three drugs together form the most promising therapeutic regimen. In a cohort of 67 metastatic patients treated for at least 12 months with vinorelbine 30 or 40 mg orally three times a week, cyclophosphamide 50 mg daily, and capecitabine 500 mg three times a day at the European Institute of Oncology, founded in 1994 by the aforementioned Umberto Veronesi, the progression-free survival at 3 years was 25.4% and at 4 years was 18.5% [93]. On the other hand, American researchers from the Seattle Cancer Care Alliance have retrospectively analyzed the impact of MCTP not in advanced disease but in early-stage breast cancer by enrolling 98 luminal A patients from February 2015 through December 2018; their study has demonstrated that MCTP with cyclophosphamide, methotrexate, and fluorouracil can offer survival outcomes equivalent to traditional chemotherapy with docetaxel and cyclophosphamide [70]. Similar results in terms of disease-free survival for high-risk early-stage breast cancer have been reported by other American researchers with doxorubicin and cyclophosphamide-based MCTP followed by weekly nab-paclitaxel [94]. In a randomized clinical trial conducted at 13 clinical and academic centers in China from April 2010 to December 2016, involving 434 women with early-stage triple-negative subtype who received standard adjuvant treatment, low-dose capecitabine maintenance therapy (650 mg twice a day orally for one year) resulted in a significant improvement in 5-year disease-free survival compared with observation (82.8% vs. 73.0%) [95]. These findings support the benefits of MCTP in triple-negative breast cancer [60,62,95]; in addition, MCTP achieves even better results when combined with anti-hormonal or targeted therapies [96,97,98].

## 4. Conclusions

In conclusion, negative SLNB means that metastasizing cells have not spread to the lymph nodes, the first organs theoretically reached in case of breast cancer dissemination; if SLNB is negative, it is very likely that the cancer has not spread to any other area of the organism. In daily practice, this assumption must be supported by a preoperative whole-body scan (e.g., computed tomography, magnetic resonance imaging). However, even in the case of negative staging, the presence of metastatic cells scattered in a para-sentinel node or in the lymphatic vessel connecting the tumor to the sentinel lymph node cannot be excluded. Therefore, pT1-T2 N0 M0 HER2-positive and ER/PR-positive breast cancer represents a further variant of the HER2-positive subtype at an early stage with a more favorable prognosis because it can be completely removed with surgery and treated with adjuvant anti-HER2 therapy, anti-hormonal therapy, and radiotherapy in order to target any residual cells in transit and not detected. In the face of this variant, MCTP would find an ideal field of application because it would help to further clean the tumor area from any residual cells while improving the response to eventual immunotherapy and preserving patients from the toxicity of conventional chemotherapy. A clinical trial for this early-stage variant with adjuvant oral MCTP after surgery in combination with targeted therapy, anti-hormonal therapy, radiotherapy, and, in case of strong PD-1/PD-L1 positivity, immunotherapy is therefore advocated.

## References

[1] Jan N., Sofi S., Qayoom H., Shabir A., Haq B.U., Macha M.A., Almilaibary A., Mir M.A. (2024). Metronomic chemotherapy and drug repurposing: A paradigm shift in oncology. Heliyon.

[2] Scharovsky O.G., Rico M.J., Mainetti L.E., Perroud H.A., Rozados V.R. (2020). Achievements and challenges in the use of metronomics for the treatment of breast cancer. Biochem. Pharmacol..

[3] Kaur H., Budd G.T. (2004). Metronomic therapy for breast cancer. Curr. Oncol. Rep..

[4] Hanahan D., Bergers G., Bergsland E. (2000). Less is more, regularly: Metronomic dosing of cytotoxic drugs can target tumor angiogenesis in mice. J. Clin. Invest..

[5] Maiti R. (2014). Metronomic chemotherapy. J. Pharmacol. Pharmacother..

[6] Lien K., Georgsdottir S., Sivanathan L., Chan K., Emmenegger U. (2013). Low-dose metronomic chemotherapy: A systematic literature analysis. Eur. J. Cancer.

[7] Pepe F.F., Cazzaniga M.E., Baroni S., Riva F., Cicchiello F., Capici S., Cogliati V., Maggioni C., Cordani N., Cerrito M.G. (2022). Immunomodulatory effects of metronomic vinorelbine (mVRL), with or without metronomic capecitabine (mCAPE), in hormone receptor positive (HR+)/HER2-negative metastatic breast cancer (MBC) patients: Final results of the exploratory phase 2 Victor-5 study. BMC Cancer.

[8] Koumarianou A., Christodoulou M.I., Patapis P., Papadopoulos I., Liakata E., Giagini A., Stavropoulou A., Poulakaki N., Tountas N., Xiros N. (2014). The effect of metronomic versus standard chemotherapy on the regulatory to effector T-cell equilibrium in cancer patients. Exp. Hematol. Oncol..

[9] Scharovsky O.G., Mainetti L.E., Rozados V.R. (2009). Metronomic chemotherapy: Changing the paradigm that more is better. Curr. Oncol..

[10] Kareva I., Waxman D.J., Lakka Klement G. (2015). Metronomic chemotherapy: An attractive alternative to maximum tolerated dose therapy that can activate anti-tumor immunity and minimize therapeutic resistance. Cancer Lett..

[11] Hao Y.B., Yi S.Y., Ruan J., Zhao L., Nan K.J. (2014). New insights into metronomic chemotherapy-induced immunoregulation. Cancer Lett..

[12] Qiao W., Hu C., Ma J., Dong X., Dalangood S., Li H., Yuan C., Lu B., Gao W.Q., Wen Z. (2023). Low-dose metronomic chemotherapy triggers oxidized mtDNA sensing inside tumor cells to potentiate CD8+T anti-tumor immunity. Cancer Lett..

[13] Mpekris F., Voutouri C., Panagi M., Baish J.W., Jain R.K., Stylianopoulos T. (2022). Normalizing tumor microenvironment with nanomedicine and metronomic therapy to improve immunotherapy. J. Control. Release.

[14] Zheng X., Kuai J., Shen G. (2023). Low-dose metronomic gemcitabine pretreatments overcome the resistance of breast cancer to immune checkpoint therapy. Immunotherapy.

[15] Roncati L. (2018). Microsatellite instability predicts response to anti-PD1 immunotherapy in metastatic melanoma. Acta Dermatovenerol. Croat..

[16] Chen Q., Xia R., Zheng W., Zhang L., Li P., Sun X., Shi J. (2020). Metronomic paclitaxel improves the efficacy of PD-1 monoclonal antibodies in breast cancer by transforming the tumor immune microenvironment. Am. J. Transl. Res..

[17] Mo H., Yu Y., Sun X., Ge H., Yu L., Guan X., Zhai J., Zhu A., Wei Y., Wang J. (2024). Metronomic chemotherapy plus anti-PD-1 in metastatic breast cancer: A Bayesian adaptive randomized phase 2 trial. Nat. Med..

[18] Pasquier E., Kavallaris M., André N. (2010). Metronomic chemotherapy: New rationale for new directions. Nat. Rev. Clin. Oncol..

[19] Tao W.Y., Liang X.S., Liu Y., Wang C.Y., Pang D. (2015). Decrease of let-7f in low-dose metronomic Paclitaxel chemotherapy contributed to upregulation of thrombospondin-1 in breast cancer. Int. J. Biol. Sci..

[20] Calleri A., Bono A., Bagnardi V., Quarna J., Mancuso P., Rabascio C., Dellapasqua S., Campagnoli E., Shaked Y., Goldhirsch A. (2009). Predictive potential of angiogenic growth factors and circulating endothelial cells in breast cancer patients receiving metronomic chemotherapy plus bevacizumab. Clin. Cancer Res..

[21] Simsek C., Esin E., Yalcin S. (2019). Metronomic chemotherapy: A systematic review of the literature and clinical experience. J. Oncol..

[22] El-Arab L.R., Swellam M., El Mahdy M.M. (2012). Metronomic chemotherapy in metastatic breast cancer: Impact on VEGF. J. Egypt Natl. Canc. Inst..

[23] Kerbel R.S. (2015). A decade of experience in developing preclinical models of advanced- or early-stage spontaneous metastasis to study antiangiogenic drugs, metronomic chemotherapy, and the tumor microenvironment. Cancer J..

[24] Aktas S.H., Akbulut H., Akgun N., Icli F. (2012). Low dose chemotherapeutic drugs without overt cytotoxic effects decrease the secretion of VEGF by cultured human tumor cells: A tentative relationship between drug type and tumor cell type response. Cancer Biomark..

[25] André N., Pasquier E. (2009). Response to ‘intermittent androgen blockade should be regarded as standard therapy in prostate cancer’. Nat. Clin. Pract. Oncol..

[26] Aguirre-Ghiso J.A. (2007). Models, mechanisms and clinical evidence for cancer dormancy. Nat. Rev. Cancer.

[27] Clements M.E., Johnson R.W. (2019). Breast cancer dormancy in bone. Curr. Osteoporos. Rep..

[28] Elkholi I.E., Lalonde A., Park M., Côté J.F. (2022). Breast cancer metastatic dormancy and relapse: An enigma of microenvironment(s). Cancer Res..

[29] Uhr J.W., Pantel K. (2011). Controversies in clinical cancer dormancy. Proc. Natl. Acad. Sci. USA.

[30] Aguirre-Ghiso J.A. (2006). The problem of cancer dormancy: Understanding the basic mechanisms and identifying therapeutic opportunities. Cell Cycle.

[31] Lenart N.A., Rao S.S. (2024). Cell-cell interactions mediating primary and metastatic breast cancer dormancy. Cancer Metastasis Rev..

[32] Naumov G.N., Akslen L.A., Folkman J. (2006). Role of angiogenesis in human tumor dormancy: Animal models of the angiogenic switch. Cell Cycle.

[33] Wikman H., Vessella R., Pantel K. (2008). Cancer micrometastasis and tumour dormancy. APMIS.

[34] Ramamoorthi G., Kodumudi K., Gallen C., Zachariah N.N., Basu A., Albert G., Beyer A., Snyder C., Wiener D., Costa R.L.B. (2022). Disseminated cancer cells in breast cancer: Mechanism of dissemination and dormancy and emerging insights on therapeutic opportunities. Semin. Cancer Biol..

[35] Cazzaniga M.E., Cordani N., Capici S., Cogliati V., Riva F., Cerrito M.G. (2021). Metronomic chemotherapy. Cancers.

[36] Global Cancer Observatory—Breast Cancer. https://gco.iarc.who.int/media/globocan/factsheets/cancers/20-breast-fact-sheet.pdf.

[37] Tanis P.J., Nieweg O.E., Valdés Olmos R.A., Th Rutgers E.J., Kroon B.B. (2001). History of sentinel node and validation of the technique. Breast Cancer Res..

[38] Veronesi U., Paganelli G., Viale G., Luini A., Zurrida S., Galimberti V., Intra M., Veronesi P., Maisonneuve P., Gatti G. (2006). Sentinel-lymph-node biopsy as a staging procedure in breast cancer: Update of a randomised controlled study. Lancet Oncol..

[39] Veronesi U., Paganelli G., Viale G., Luini A., Zurrida S., Galimberti V., Intra M., Veronesi P., Robertson C., Maisonneuve P. (2003). A randomized comparison of sentinel-node biopsy with routine axillary dissection in breast cancer. N. Engl. J. Med..

[40] Piscioli F., Pusiol T., Roncati L. (2017). Wisely choosing thin melanomas for sentinel lymph node biopsy. J. Am. Acad. Dermatol..

[41] Hortobagyi G.N., Connolly J.L., D’Orsi C.J., Edge S.B., Mittendorf E.A., Rugo H.S., Solin L.J., Weaver D.L., Winchester D.J., Giuliano A., Amin M.B., Edge S.B., Greene F.L., Byrd D.R., Brookland R.K., Washington M.K., Gershenwald J.E., Compton C.C., Hess K.R., Sullivan D.C. (2017). Breast. AJCC Cancer Staging Manual.

[42] Curtaz C.J., Kiesel L., Meybohm P., Wöckel A., Burek M. (2022). Anti-hormonal therapy in breast cancer and its effect on the blood-brain barrier. Cancers.

[43] Trayes K.P., Cokenakes S.E.H. (2021). Breast cancer treatment. Am. Fam. Physician.

[44] American Cancer Society—Chemotherapy for Breast Cancer. https://www.cancer.org/content/dam/CRC/PDF/Public/8581.00.pdf.

[45] Harbeck N., Beckmann M.W., Rody A., Schneeweiss A., Müller V., Fehm T., Marschner N., Gluz O., Schrader I., Heinrich G. (2013). HER2 dimerization inhibitor pertuzumab—Mode of action and clinical data in breast cancer. Breast Care.

[46] Clynes R.A., Towers T.L., Presta L.G., Ravetch J.V. (2000). Inhibitory Fc receptors modulate in vivo cytotoxicity against tumor targets. Nat. Med..

[47] Lehmann B.D., Bauer J.A., Chen X., Sanders M.E., Chakravarthy A.B., Shyr Y., Pietenpol J.A. (2011). Identification of human triple-negative breast cancer subtypes and preclinical models for selection of targeted therapies. J. Clin. Invest..

[48] Núñez Abad M., Calabuig-Fariñas S., Lobo de Mena M., Torres-Martínez S., García González C., García García J.Á., Iranzo González-Cruz V., Camps Herrero C. (2022). Programmed death-ligand 1 (PD-L1) as immunotherapy biomarker in breast cancer. Cancers.

[49] Liu J., He M., Wang Z., Li Q., Xu B. (2022). Current research status of metronomic chemotherapy in combination treatment of breast cancer. Oncol. Res. Treat..

[50] Krajnak S., Battista M.J., Hasenburg A., Schmidt M. (2022). Metronomic chemotherapy for metastatic breast cancer. Oncol. Res. Treat..

[51] Liu Y., Gu F., Liang J., Dai X., Wan C., Hong X., Zhang K., Liu L. (2017). The efficacy and toxicity profile of metronomic chemotherapy for metastatic breast cancer: A meta-analysis. PLoS ONE.

[52] Krajnak S., Schnatz C., Almstedt K., Brenner W., Haertner F., Heimes A.S., Lebrecht A., Makris G.M., Schwab R., Hasenburg A. (2020). Low-dose metronomic chemotherapy as an efficient treatment option in metastatic breast cancer-results of an exploratory case-control study. Breast Cancer Res. Treat..

[53] Kontani K., Hashimoto S.I., Murazawa C., Norimura S., Tanaka H., Ohtani M., Fujiwara-Honjo N., Date M., Teramoto K., Houchi H. (2016). Indication of metronomic chemotherapy for metastatic breast cancer: Clinical outcomes and responsive subtypes. Mol. Clin. Oncol..

[54] Liu J., Zhang J., Li H., Song G., Di L., Jiang H., Yan Y., Wang H., Wang J., Liu X. (2025). Anlotinib in combination with metronomic chemotherapy in HER2-negative metastatic breast cancer: An observational and retrospective study. BMC Cancer.

[55] Azim H.A., Saleh M.A., Essam Eldin P., Abdelhafeez A.A.M., Hassan M., Kassem L. (2025). Combination of metronomic capecitabine and letrozole in metastatic hormone receptor positive, HER2 negative breast cancer: A randomized phase II trial. J. Chemother..

[56] Chai Y., Liu J., Jiang M., He M., Wang Z., Ma F., Wang J., Yuan P., Luo Y., Xu B. (2023). A phase II study of a doublet metronomic chemotherapy regimen consisting of oral vinorelbine and capecitabine in Chinese women with HER2-negative metastatic breast cancer. Thorac. Cancer.

[57] Hao C., Wang X., Shi Y., Tong Z., Li S., Liu X., Zhang L., Zhang J., Meng W., Zhang L. (2024). Combination therapy of pyrotinib and metronomic vinorelbine in HER2+ advanced breast cancer after trastuzumab failure (PROVE): A prospective phase 2 study. Cancer Res. Treat..

[58] He M., Liu J., Wang Z., Ma F., Wang J., Zhang P., Li Q., Yuan P., Luo Y., Fan Y. (2023). Safety and efficacy study of oral metronomic capecitabine combined with pyrotinib in HER2-positive metastatic breast cancer: A phase II trial. Breast.

[59] Chi Y., Shang M., Xu L., Gong H., Tao R., Song L., Zhang B., Yin S., Cong B., Li H. (2022). Durable effect of pyrotinib and metronomic vinorelbine in HER2-positive breast cancer with leptomeningeal disease: A case report and literature review. Front. Oncol..

[60] Alagizy H.A., Shehata M.A., Hashem T.A., Abdelaziz K.K., Swiha M.M. (2015). Metronomic capecitabine as extended adjuvant chemotherapy in women with triple negative breast cancer. Hematol. Oncol. Stem Cell. Ther..

[61] Buda-Nowak A., Kwinta Ł., Potocki P., Michałowska-Kaczmarczyk A., Słowik A., Konopka K., Streb J., Koniewski M., Wysocki P.J. (2023). Metronomic chemo-endocrine therapy (FulVEC) as a salvage treatment for patients with advanced, treatment-refractory ER+/HER2-breast cancer-a retrospective analysis of consecutive patients data. J. Clin. Med..

[62] Colleoni M., Gray K.P., Gelber S., Láng I., Thürlimann B., Gianni L., Abdi E.A., Gomez H.L., Linderholm B.K., Puglisi F. (2016). Low-dose oral cyclophosphamide and methotrexate maintenance for hormone receptor-negative early breast cancer: International breast cancer study group trial 22-00. J. Clin. Oncol..

[63] Orlando L., Lorusso V., Giotta F., Di Maio M., Schiavone P., Fedele P., Quaranta A., Caliolo C., Ciccarese M., Cinefra M. (2020). Metronomic oral chemotherapy with cyclophosphamide plus capecitabine combined with trastuzumab (HEX) as first line therapy of HER-2 positive advanced breast cancer: A phase II trial of the Gruppo Oncologico Italia Meridionale (GOIM). Breast.

[64] Hong R.X., Xu F., Xia W., Teng Y.E., Ouyang Q.C., Zheng Q.F., Yuan Z.Y., Chen D.S., Jiang K.K., Lin Y. (2025). Metronomic capecitabine plus aromatase inhibitor as initial therapy in patients with hormone receptor-positive, human epidermal growth factor receptor 2-negative metastatic breast cancer-the phase III MECCA trial. J. Clin. Oncol..

[65] Mutlu H., Musri F.Y., Artaç M., Kargi A., Özdogan M., Bozcuk H. (2015). Metronomic oral chemotherapy with old agents in patients with heavily treated metastatic breast cancer. J. Cancer Res. Ther..

[66] Wildiers H., Tryfonidis K., Dal Lago L., Vuylsteke P., Curigliano G., Waters S., Brouwers B., Altintas S., Touati N., Cardoso F. (2018). Pertuzumab and trastuzumab with or without metronomic chemotherapy for older patients with HER2-positive metastatic breast cancer (EORTC 75111-10114): An open-label, randomised, phase 2 trial from the Elderly Task Force/Breast Cancer Group. Lancet Oncol..

[67] Manso L., Valdiviezo N., Sepúlveda J., Ciruelos E., Mendiola C., Ghanem I., Vega E., Manneh R., Dorta M., Cortés-Funes H. (2013). Safety and efficacy of metronomic non-pegylated liposomal encapsulated doxorubicin in heavily pretreated advanced breast cancer patients. Clin. Transl. Oncol..

[68] Fedele P., Marino A., Orlando L., Schiavone P., Nacci A., Sponziello F., Rizzo P., Calvani N., Mazzoni E., Cinefra M. (2012). Efficacy and safety of low-dose metronomic chemotherapy with capecitabine in heavily pretreated patients with metastatic breast cancer. Eur. J. Cancer.

[69] Perroud H.A., Alasino C.M., Rico M.J., Queralt F., Pezzotto S.M., Rozados V.R., Scharovsky O.G. (2016). Quality of life in patients with metastatic breast cancer treated with metronomic chemotherapy. Future Oncol..

[70] Jung L., Miske A., Indorf A., Nelson K., Gadi V.K., Banda K. (2022). A retrospective analysis of metronomic cyclophosphamide, methotrexate, and fluorouracil (CMF) versus docetaxel and cyclophosphamide (TC) as adjuvant treatment in early stage, hormone receptor positive, HER2 negative breast cancer. Clin. Breast Cancer.

[71] Nasr K.E., Osman M.A., Elkady M.S., Ellithy M.A. (2015). Metronomic methotrexate and cyclophosphamide after carboplatin included adjuvant chemotherapy in triple negative breast cancer: A phase III study. Ann. Transl. Med..

[72] Lu Q., Lee K., Xu F., Xia W., Zheng Q., Hong R., Jiang K., Zhai Q., Li Y., Shi Y. (2020). Metronomic chemotherapy of cyclophosphamide plus methotrexate for advanced breast cancer: Real-world data analyses and experience of one center. Cancer Commun..

[73] Abdelmaksoud B.A., Mohammed A., Toam M.M. (2020). A pilot study of extended adjuvant therapy with metronomic docetaxel for patients with operable triple-negative breast cancer. Asian Pac. J. Cancer Prev..

[74] Korantzis I., Kalogeras K.T., Papaxoinis G., Kotoula V., Koutras A., Soupos N., Papakostas P., Dionysopoulos D., Samantas E., Christodoulou C. (2012). Expression of angiogenic markers in the peripheral blood of patients with advanced breast cancer treated with weekly docetaxel. Anticancer Res..

[75] Español A.J., Salem A., Di Bari M., Cristofaro I., Sanchez Y., Tata A.M., Sales M.E. (2020). The metronomic combination of paclitaxel with cholinergic agonists inhibits triple negative breast tumor progression. Participation of M2 receptor subtype. PLoS ONE.

[76] Crivellari D., Gray K.P., Dellapasqua S., Puglisi F., Ribi K., Price K.N., Láng I., Gianni L., Spazzapan S., Pinotti G. (2013). Adjuvant pegylated liposomal doxorubicin for older women with endocrine nonresponsive breast cancer who are not suitable for a “standard chemotherapy regimen”: The CASA randomized trial. Breast.

[77] Chalasani P., Farr K., Wu V., Jenkins I., Liu A., Parker S., Gadi V.K., Specht J., Linden H. (2021). Single arm, phase two study of low-dose metronomic eribulin in metastatic breast cancer. Breast Cancer Res. Treat..

[78] Chen X., He Y., Fan T., Wei Y. (2025). Efficacy and safety of low-dose oral etoposide combined with capecitabine for patients with postoperative metastatic breast cancer resistant to anthracycline/taxanes. Thorac. Cancer.

[79] Liu J., He M., Jiang M., Zhou S., Zhang M., Li Y., Chen S., Cai R., Mo H., Lan B. (2024). Pyrotinib combined with metronomic etoposide in heavily pretreated HER2-positive metastatic breast cancer: A single-arm, phase II study. BMC Cancer.

[80] Jenkins S., Zhang W., Steinberg S.M., Nousome D., Houston N., Wu X., Armstrong T.S., Burton E., Smart D.D., Shah R. (2023). Phase I study and cell-free DNA analysis of T-DM1 and metronomic temozolomide for secondary prevention of HER2-positive breast cancer brain metastases. Clin. Cancer Res..

[81] Liu C.T., Hsieh M.C., Su Y.L., Hung C.M., Pei S.N., Liao C.K., Tsai Y.F., Liao H.Y., Liu W.C., Chiu C.C. (2021). Metronomic vinorelbine is an excellent and safe treatment for advanced breast cancer: A retrospective, observational study. J. Cancer.

[82] Wang Z., Liu J., Ma F., Wang J., Luo Y., Fan Y., Yuan P., Zhang P., Li Q., Li Q. (2021). Safety and efficacy study of oral metronomic vinorelbine combined with trastuzumab (mNH) in HER2-positive metastatic breast cancer: A phase II trial. Breast Cancer Res. Treat..

[83] Sanna G., Pestrin M., Moretti E., Biagioni C., De Santo I., Gabellini S., Galardi F., McCartney A., Biganzoli L. (2021). A dose-finding study of metronomic oral vinorelbine in combination with oral cyclophosphamide and bevacizumab in patients with advanced breast cancer. Clin. Breast Cancer.

[84] Adamo B., Bellet M., Paré L., Pascual T., Vidal M., Pérez Fidalgo J.A., Blanch S., Martinez N., Murillo L., Gómez-Pardo P. (2019). Oral metronomic vinorelbine combined with endocrine therapy in hormone receptor-positive HER2-negative breast cancer: SOLTI-1501 VENTANA window of opportunity trial. Breast Cancer Res..

[85] Delahousse J., Molina L., Paci A. (2024). Cyclophosphamide and analogues; a matter of dose and schedule for dual anticancer activities. Cancer Lett..

[86] Anampa J., Chen A., Wright J., Patel M., Pellegrino C., Fehn K., Sparano J.A., Andreopoulou E. (2018). Phase I trial of veliparib, a poly ADP ribose polymerase inhibitor, plus metronomic cyclophosphamide in metastatic HER2-negative breast cancer. Clin. Breast Cancer..

[87] Muñoz R., Hileeto D., Cruz-Muñoz W., Wood G.A., Xu P., Man S., Viloria-Petit A., Kerbel R.S. (2019). Suppressive impact of metronomic chemotherapy using UFT and/or cyclophosphamide on mediators of breast cancer dissemination and invasion. PLoS ONE.

[88] Bazzola L., Foroni C., Andreis D., Zanoni V., Cappelletti M.R., Allevi G., Aguggini S., Strina C., Milani M., Venturini S. (2015). Combination of letrozole, metronomic cyclophosphamide and sorafenib is well-tolerated and shows activity in patients with primary breast cancer. Br. J. Cancer.

[89] Wang Z., Lu J., Leaw S., Hong X., Wang J., Shao Z., Hu X. (2012). An all-oral combination of metronomic cyclophosphamide plus capecitabine in patients with anthracycline- and taxane-pretreated metastatic breast cancer: A phase II study. Cancer Chemother. Pharmacol..

[90] Larsson K.F., Adra J., Klint L., Linderholm B. (2024). Metronomic chemotherapy using capecitabine and cyclophosphamide in metastatic breast cancer—Efficacy, tolerability and quality of life results from the phase II METRO trial. Breast.

[91] Yoshimoto M., Takao S., Hirata M., Okamoto Y., Yamashita S., Kawaguchi Y., Takami M., Furusawa H., Morita S., Abe C. (2012). Metronomic oral combination chemotherapy with capecitabine and cyclophosphamide: A phase II study in patients with HER2-negative metastatic breast cancer. Cancer Chemother. Pharmacol..

[92] Li J.W., Zuo W.J., Ivanova D., Jia X.Q., Lei L., Liu G.Y. (2019). Metronomic capecitabine combined with aromatase inhibitors for new chemoendocrine treatment of advanced breast cancer: A phase II clinical trial. Breast Cancer Res. Treat..

[93] Montagna E., Pagan E., Cancello G., Sangalli C., Bagnardi V., Munzone E., Salè E.O., Malengo D., Cazzaniga M.E., Negri M. (2022). The prolonged clinical benefit with metronomic chemotherapy (VEX regimen) in metastatic breast cancer patients. Anticancer Drugs.

[94] Cho E., Wu Q., Rubinstein L., Linden H., Gralow J., Specht J., Gadi V., Ellis G. (2018). Adjuvant continuous metronomic adriamycin + cyclophosphamide followed by weekly nab-paclitaxel for high-risk early-stage breast cancer. Breast J..

[95] Wang X., Wang S.S., Huang H., Cai L., Zhao L., Peng R.J., Lin Y., Tang J., Zeng J., South China Breast Cancer Group (SCBCG) (2021). Effect of capecitabine maintenance therapy using lower dosage and higher frequency vs observation on disease-free survival among patients with early-stage triple-negative breast cancer who had received standard treatment: The SYSUCC-001 randomized clinical trial. JAMA.

[96] Trapani D., Jin Q., Miller K.D., Rugo H.S., Reeder-Hayes K.E., Traina T., Abdou Y., Falkson C., Abramson V., Ligibel J. (2024). Optimizing postneoadjuvant treatment of residual breast cancer with adjuvant bevacizumab alone, with metronomic or standard-dose chemotherapy: A combined analysis of DFCI 05-055 and DFCI 09-134/TBCRC 012/ABCDE clinical trials. Clin. Breast Cancer.

[97] Mayer E.L., Tayob N., Ren S., Savoie J.J., Spigel D.R., Burris H.A., Ryan P.D., Harris L.N., Winer E.P., Burstein H.J. (2024). A randomized phase II study of metronomic cyclophosphamide and methotrexate (CM) with or without bevacizumab in patients with advanced breast cancer. Breast Cancer Res. Treat..

[98] García-Sáenz J.A., Martín M., Calles A., Bueno C., Rodríguez L., Bobokova J., Custodio A., Casado A., Díaz-Rubio E. (2008). Bevacizumab in combination with metronomic chemotherapy in patients with anthracycline- and taxane-refractory breast cancer. J. Chemother..

